# High-risk HPV is not associated with epithelial ovarian cancer in a Caucasian population

**DOI:** 10.1186/s13027-016-0087-4

**Published:** 2016-07-14

**Authors:** Kasper Ingerslev, Estrid Hogdall, Wojciech Skovrider-Ruminski, Tine Henrichsen Schnack, Mona Aarenstrup Karlsen, Lotte Nedergaard, Claus Hogdall, Jan Blaakær

**Affiliations:** Department of Gynaecology and Obstetrics, Aarhus University Hospital, Palle Juul-Jensens Blvd. 99, 8200 Aarhus N, Denmark; Department of Pathology, Herlev University Hospital, Herlev Ringvej 75, 2730 Herlev, Denmark; Department of Gynaecology and Obstetrics, Copenhagen University Hospital, Rigshospitalet, Blegdamsvej 9, 2100 København Ø, Denmark; Molecular Unit, Department of Pathology, Herlev University Hospital & Gynecologic clinic, Copenhagen University Hospital, Rigshospitalet, København Ø, Denmark; Department of Pathology, Copenhagen University Hospital, Rigshospitalet, Blegdamsvej 9, 2100 København Ø, Denmark

**Keywords:** Human papillomavirus, Ovarian cancer, Viral carcinogenesis

## Abstract

**Background:**

High-risk human papillomavirus (HPV) has been suspected to play a role in the carcinogenesis of epithelial ovarian cancer (EOC). However, results from previous studies are conflicting. In most of these studies, the number of tissue samples was small. The current study was therefore undertaken to examine the prevalence of high-risk HPV DNA in EOC in a large series of patients.

**Method:**

Formalin-fixed, paraffin-imbedded tumor tissue samples from 198 cases consecutively included in the Danish Pelvic Mass Study were analyzed. The material included 163 serous adenocarcinomas, 15 endometrioid adenocarcinomas, 11 mucinous adenocarcinomas and nine clear-cell carcinomas. Genotyping for high-risk HPV DNA was performed by real-time Polymerase chain reaction (PCR) using an in-house TaqMan singleplex assay targeting the E6/E7 region of the HPV 16 and 18 genomes. Additionally, 20 random samples without HPV 16 and/or 18 infections were reanalyzed for HPV subtypes 31, 33, 35, 39, 45, 51 and 52.

**Results:**

The quality criteria were fulfilled in 191 samples. HPV 18 DNA was detected in one sample only, while the rest tested negative. The subgroup analysis for seven additional high-risk HPV subtypes was also negative.

**Conclusions:**

Only one in 191 samples was positive for HPV DNA. We therefore conclude that high risk HPV is unlikely to be associated with EOC in a Caucasian population. Future studies should focus on other microorganisms as possible etiological factors in EOC carcinogenesis.

**Electronic supplementary material:**

The online version of this article (doi:10.1186/s13027-016-0087-4) contains supplementary material, which is available to authorized users.

## Background

Epithelial ovarian cancer (EOC) is the most lethal cancer of all the gynecological cancers. It is characterized by late and unspecific onset of symptoms and thus the presence of disseminated disease at the time of diagnosis [1]. Ten percent of EOC cases are estimated to be caused by genetic mutations, most notably BRCA1 and BRCA2 mutations [2]. An inverse association with the number of ovulatory cycles has also been postulated due to the protective effect of parity and oral contraceptives on EOC risk [3]. Despite of these findings, the etiology of EOC is still largely unknown. A current theory hypothesizes that pelvic infection and inflammation may play a role in the carcinogenesis of EOC [4, 5]. The theory is supported by epidemiological data that describes a protective effect on EOC of factors that interrupt the passage to the peritoneal cavity from the vagina. This applies, for instance, to tubal ligation, salpingectomy and hysterectomy [6–8]. Due to the known association between human papillomavirus (HPV) and cervical, vaginal, vulvar, anal and oropharyngeal cancers, the focus has primarily been directed toward high-risk HPV [9, 10]. However, previous data are conflicting. Several studies have found an association between HPV and EOC [11–14], whereas others have not [15–18]. Thus, the possible association remains unclear. Previous studies are characterized by a small number of included patients with EOC, resulting in limited statistical power. Additionally, not all studies stratified their analysis by EOC subtype. This stratification is important since EOC subtypes are believed to develop through different pathogenetic pathways [19, 20], and therefore, it may be assumed that the etiologic factors differ among subtypes. The current study was therefore undertaken to determine the prevalence of high-risk HPV DNA in EOC tissue from a large group of patients. We used real-time PCR assays on DNA extracted from 198 tumor tissue samples from EOC patients. Full pathology information was obtained for all patients, allowing positive results to be correlated with EOC subtype.

## Material

The study was a part of the Danish Pelvic Mass Study, a prospective, ongoing collection of blood and tumor tissue samples. Oral and written consent were given by each patient before enrollment, and the Danish Ethical Committee approved the study protocol (KF01-227/03 and KF01-143/04, H-3-2010-022). Ninety-five percent of patients that were eligible for inclusion accepted to participate. In the period from 2004–2010, 246 patients with EOC were consecutively included. The study was a single center study (Copenhagen University Hospital) in the first few years, accounting for the relatively low rate of inclusion. The study has since expanded to be nation-wide. The patients were included when admitted to the tertiary center, the Gynecologic Clinic, Copenhagen University Hospital, Copenhagen, Denmark due to a pelvic mass or pelvic pains potentially caused by EOC. Patients with preoperatively known relapse of previous cancer or an active cancer other than EOC were excluded. Forty-eight patients were excluded for the following reasons: 24 patients were excluded due to insufficient tumor tissue for analysis. Fifteen patients were excluded since they had been treated with neoadjuvant chemotherapy. An additional eight patients were excluded after pathology revision, since two patients did not have EOC, four patients had carcinosarcomas and two patients had an additional cancer diagnosis. Patients were examined according to the Danish Cancer Fast Track Guidelines. All patient data were registered continuously online in the Danish Gynecologic Cancer Database by gynecologists, pathologists and oncologists. Biological material was collected and stored at the time of surgery through the Danish Cancer Biobank [21]. For the present study, formalin-fixed and paraffin-embedded (FFPE) EOC tumor samples from 198 consecutively included patients were used.

## Methods

A pathologist, specialized in gynecological oncology, revised tumor FFPE material and a 2-mm biopsy punch was then used to sample the tumor tissue. DNA extraction was carried out on a Qiagen® Qiacube using the DNA FFPE kit. DNA concentration was measured on Nanodrop® and diluted to 5 ng/μl. Genotyping was performed with an in-house singleplex assay based on published studies by Lindh et al. [22] for subtypes 16 and 18. The sensitivity of the assay was not specified in the original article [22]. However, the method was validated by the group against a linear array from Roche with high concordance. We have used the assay previously and have successfully detected a HPV DNA prevalence under one percent [23]. We therefore estimate that the sensitivity is below one percent. The assay consisted of TaqMan singleplex real-time PCR targeting the E6/E7 region of the HPV genome. Probes and corresponding primers were chosen with specificity for each HPV genotype using a housekeeping glyceraldehyde-3-phosphate dehydrogenase (GAPDH) as positive DNA reference. Additionally, 20 random samples without HPV 16 and 18 infections were further analyzed with an in-house singleplex assay for detection of HPV subtypes 31, 33, 35, 39, 45, 51 and 52 based on the method designed by Lindh et al. [22]. Real-time PCR was performed on the Roche®LC480 Lightcycler with the following cycling parameters: 50 °C for 5 min, 95 °C for 10 min followed by 50 cycles at 95 °C for 15 s, 58 °C for 1 min. A cycle threshold < 35 was used in the interpretation of the PCR results according to the recommendations in the original assay design. Each PCR reaction included 0.5 μM primer and 0.2 μM probe in 25 μl volume. Additionally, 12.5 μl TaqMan universal mastermix with Uracil-DNA Glycosylase including 5 μl sample template was added per well. There were two negative controls. A no template control (NTC) and a wildtype human DNA (TaqMan® Control Genomic DNA (human) Catalog number: 4312660). Controls were included in all runs.

## Results

**Fig. 1:**
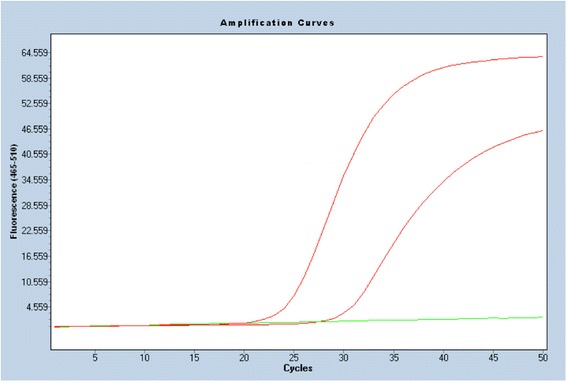
Results of real-time PCR amplification. Amplification curve showing a positive detection of HPV 18 DNA and housekeeping gene GAPDH. HPV 16 in negative

The tissue material included in this study consisted of 163 serous adenocarcinomas (82.3 %), 15 endometrioid adenocarcinomas (7.6 %), 11 mucinous adenocarcinomas (5.5 %) and nine clear cell neoplasms (4.5 %). The International Federation of Gynecology and Obstetrics (FIGO) stage distribution of the study population was as follows: 31 had stage I EOC (15.7 %), 20 patients had stage II (10.1 %), 122 had stage III (61.6 %) and 25 had stage IV EOC (12.6 %). The median age was 64 years (range 31–89 years). No differences in age, stage and distribution of histology were found between the 198 included patients with EOC and the 24 patients with EOC excluded due to insufficient tumor tissue. Please be referred to Table 1. The quality criteria for the real-time PCR analyses were fulfilled in 191 samples; seven samples yielded a cycle threshold score above 35 and were excluded due to low amounts of DNA material. HPV 18 DNA was detected in one of the 191 samples (Fig. 1). The HPV-positive tumor sample was a serous adenocarcinoma, FIGO stage IB. The patient was 57 years old at the time of diagnosis. Serum CA125 was 96 U/mL. None of the tumor samples were HPV 16 positive. Furthermore, none of the 20 samples analyzed for HPV subtypes 31, 33, 35, 39, 45, 51 and 52 tested positive.

**Table 1:** Histology, stage and age of included vs. excluded patients

	EOC included *N* = 198	EOC excluded due to insufficient tumor tissue *N* = 24	*P-*Value
Histology			0.189
*Serous adenocarcinoma, n (%)*	163(82.3 %)	17(70.8 %)	
*Mucinous adenocarcinoma, n* (%)	11(5.6 %)	1(4.2 %)	
*Endometrioid adenocarcinoma, n* (%)	15(7.6 %)	3(12.5 %)	
*Clear Cell carcinoma, n (%)*	9(4.5 %)	3(12.5 %)	
Tumor stage			0.494
Stage I, *n* (%)	31(15.7 %)	6(25.0)	
Stage II, *n* (%)	21(10.6 %)	1(4.2 %)	
Stage III, *n* (%)	120(60.1 %)	13(54.2 %)	
Stage IV, *n* (%)	26(13.1 %)	4(16.7 %)	
Age, median (range)	64(58)	62(53)	0.414

Abbreviations: *EOC* epithelial ovarian cancer, *Tumor stage* the international federation of gynecology and obstetrics (FIGO) stage

## Discussion

The carcinogenic potential of high-risk HPV is well described and includes several mechanisms. Viral oncogenes E6 and E7 are pivotal elements, since expression of these genes impairs the function of host-cell tumor suppressors p53 and retinoblastoma protein, thus favoring malignant transformation [23, 24]. Furthermore, molecular studies have demonstrated the capability of E6 and E7 to target cellular factors like paxillin, tuberin, E6-AP, E6-BP, E6TP1 and TNFR-1 as well as cell cycle regulators such as cyclins, cyclin-dependent kinases and CDKs-inhibitors [24–26]. The resulting suppression of apoptosis and the prolongation of the cell life span maximize HPV DNA replication in the infected cell. High-risk HPV is an established etiological factor in anogenital and oropharyngeal cancers [9, 10]. The role of HPV in the carcinogenesis of EOC is controversial. Our study indicates that HPV is unlikely to be involved in EOC carcinogenesis in the Danish population as only one of 191 DNA sample was HPV 18 positive and none were positive for HPV 16. Furthermore, additional testing for several other HPV subtypes confirmed that these HPV subtypes were also found to be negative. This is in line with several previous studies, although results are conflicting (Table 2) [11, 12, 15, 16, 18, 26–35].

**Table 2:** Studies published during the last 20 years, reporting on HPV prevalence in EOC tissue

Author and publication year	Country of origin	Tissue type	Detection method	HPV genotypes	Number of cases of EOC	Number of HPV positive cases	HPV prevalence (%)
Asia					247	81	32.8
Shanmughapriya et al. 2012 [14]	India	Fresh	PCR	6	24	6	25
Atalay et al. 2007 [30]	Turkey	FFPE	PCR	16,33	94	8	8.5
Kuscu et al. 2005 [57]	Turkey	FFPE	In situ hybridization	Not specified	40	15	37.5
Wu et al. 2003 [13]	China	FFPE	In situ hybridization	16,18	50	26	52
Li et al. 2002 [33]	China	FFPE	PCR	Not specified	39	26	66.7
Europe, Western					274	1	0.4
Idahl et al. 2010 [18]	Sweden	Fresh frozen	PCR		52	0	0
Giordano et al. 2008 [12]	Italy	FFPE	PCR	Not specified	50	1	2
Wentzensen et al. 2008 [29]	Germany	FFPR	PCR		74	0	0
Anttila et al. 1999 [15]	Finland	FFPE	PCR		98	0	0
Europe, Eastern					71	16	22.5
Bilyk et al. 2011 [28]	Ukraine	FFPE	PCR	16,18	53	9	17
Zimna et al. 1997 [35]	Poland	Fresh frozen	PCR	18	18	7	38.9
Middle East					100	42	42
Al-Shabanah et al. 2013 [11]	Saudi Arabia	FFPE	PCR	16,18,45	100	42	42
USA					36	0	0
Quirk et al. 2006 [16]	USA	Fresh frozen	PCR		16	0	0
Chen et al. 1999 [34]	USA	FFPE	PCR		20	0	0

Abbreviations: *EOC* epithelial ovarian cancer, *PCR* polymerase chain reaction, *HPV* human papillomavirus, *FFPE* formalin-fixed paraffin-embedded

The strength of the present study was the large number of samples compared to previous studies. In addition, the detailed description of the characteristics of the patients, including information on tumor histology and FIGO stage, should be noted. Some limitations should also be addressed. Indeed, HPV DNA detection may be less optimal in FFPE tumor material compared to fresh frozen tissue due to nucleic acid degradation [35, 36]. Recently we have, however, successfully demonstrated HPV DNA in 90 % of anal cancers using a comparable FFPE material in the same laboratory [23, 36]. Moreover, the previous studies with the highest reported HPV prevalences used FFPE material [11, 13,32, 33] (Table 2). Our study did not include a control group. However, since we report a very low prevalence of HPV DNA, it is our opinion that a control group would not alter the conclusions of the study. Another limitation is that signs of HPV infection may be lost in the interval between the primary infection and the diagnosis of cancer. For comparison, the time between primary HPV infection and the development of cancer of the cervix uteri has been estimated to be 15–20 years [37]. However, in other HPV-related cancers, such as cervical and anal cancer, viral DNA is present in the malignant tumor tissue and not exclusively in the precancerous lesions [38, 39]. Moreover, continuous expression of viral oncogenes is considered necessary for the sustenance of the malignant phenotype in cancers associated with HPV [40]. In the event that HPV is involved in the pathogenesis of EOC, we would therefore expect that HPV DNA would still be present. A number of aspects could account for the conflicting results of the available studies on EOC and HPV. Firstly, different analyzing methods were used. For instance, Wu et al. used both In situ hybridization and immunohistochemistry on the same 50 FFPE EOC samples and detected HPV 16 DNA in 52 % and 36 % of samples, respectively [13]. In addition, differences in race or country of origin may play a role. Studies from Asia generally report higher prevalence of HPV in EOC tumor tissues than studies from the Western countries [35, 36]. This is in support of our findings. Several factors could account for this difference. Firstly, HPV may be more prevalent in some regions of the World and most notably in the developing countries [41]. Another factor could be the distribution of more virulent high-risk HPV strains in Asia [42], or that the Asian populations have a higher genetic susceptibility to HPV-induced carcinogenesis, e.g. through expression of certain variants of polymorphisms like the *TNFA*-308G/A (rs1800629) and -238G/A (rs361525) or the p53 codon 72 polymorphism [43]. Despite the high Asian prevalence of HPV in EOC tissue, the overall prevalence of ovarian cancer is lower in Asian countries compared to Western countries [44]. This speaks against a connection between EOC and HPV even though the etiology of EOC is most likely multi-factorial.

Our results do not support the theory that HPV is associated with EOC. However, other microorganisms may play a role in the development of EOC. Thus, pelvic inflammatory disease (PID) has been associated with an increased risk of EOC in some epidemiological studies that also report a dose–response effect, with more episodes of PID associated with a stronger risk of EOC [45, 46]. Other studies, however, have not confirmed this association [47–49]. Inflammatory cells can promote neoplastic transformation by the induction of angiogenesis, invasion and metastasis and through the release of mutagenic reactive oxygen species [50]. If an association between EOC and PID exists, it is therefore still unclear whether the microorganisms involved are directly carcinogenic or indirectly promotes a carcinogenic microenvironment by inducing tissue inflammation. Indeed, inflammation is also a key point in the incessant ovulation theory that is characterized by repetitive damage, resulting in inflammation in the ovarian surface epithelium [33]. Pelvic inflammation is also a characteristic of endometriosis, and the condition has been associated with increased risk of especially clear-cell and endometrioid carcinomas [51]. However, a direct or combined mechanism is also possible since in vitro models have demonstrated a carcinogenic potential of common bacterial and viral pathogens involved in PID [52–54]. Serous EOC is suspected to originate in the precursor lesions in the distal fallopian tubes [55], and the tubes are often affected by PID [56]. Therefore, neoplastic or precancerous lesions from the fallopian tubes may be more suitable candidates for future studies on EOC and PID. Conclusively, the role of PID in EOC is still controversial, and more studies including a broader range of microorganisms are warranted.

## Conclusion

The present comprehensive study of 198 patients with EOC does not support an association between high-risk HPV infection and EOC. It confirms the findings from previous, smaller, studies that also report no or little association in Western countries. Future studies examining the role of infectious agents involved in the pathogenesis of EOC should focus on other microorganisms as possible etiological factors in EOC carcinogenesis.

## Abbreviations

EOC, epithelial ovarian cancer; FFPE, formalin-fixed and paraffin-embedded; FIGO, international federation of gynecology and obstetrics; GAPDH, glyceraldehyde-3-phosphate dehydrogenase; HPV, human papillomavirus; PCR, polymerase chain reaction; PID, pelvic inflammatory disease

## Additional file

Additional file 1: Dataset containing information on individual samples and PCR reactions. (XLSM 26 kb)
